# Supplementary material for: Identification of photoperiod-induced specific miRNAs in the adrenal glands of Sunite sheep (Ovis aries)
Source: Front Vet Sci. 2022 Jul 22;9:888207. doi: 10.3389/fvets.2022.888207 (PMC9354845; doi:10.3389/fvets.2022.888207)
Supplement: Supplementary file 1 [file Data_Sheet_1.ZIP › Supplementary file/miRDeep2 file.zip!/miRDeep2 file/Know_Structure/SPLP42A1/oar-mir-19b.pdf]

miRBase precursor	: oar-mir-19b
Total read count	: 2103
oar-miR-19b read count	: 2099
remaining reads	: 4

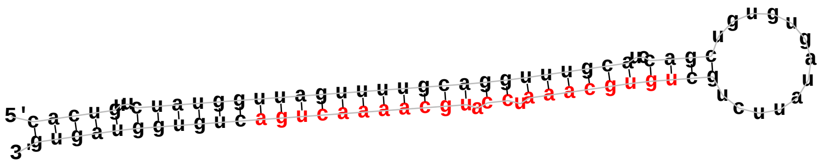

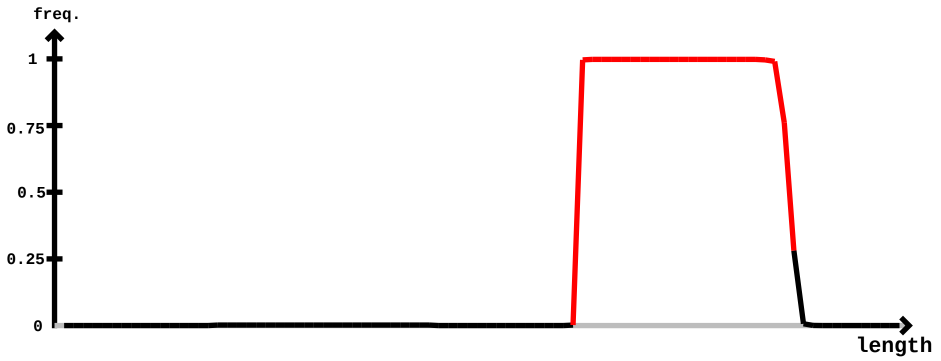

oar-miR-19b

5'- cacuguucuaugguuaguuuugcagguuugcauccagcugugugauauucugcugugcaaauccaugcaaaacugacugugguagug -3'	exp		
(((((..(((((((((((((((((((((((((..((((.............)))))))))).)).))))))))))))))))))))))	reads	mm	sample
...............aguuuugcagguuugcauccagc...	4	0	seq
....cugugcaaauccaugcaaaacu.............	1	0	seq
....cugugcaaauccaugcaaaacug............	1	0	seq
....cugugcaaauccaugcaaaacuga...........	2	0	seq
....ugugcaaauccaugcaaaa...............	4	0	seq
....ugugcaaauccaugcaaaac..............	12	0	seq
....ugugcaaauccaugcaaaacu.............	421	0	seq
....ugugcaaaucUaugcaaaacu.............	62	1	seq
....ugugcaaauccaugcaaaacug............	913	0	seq
....ugugcaaaucUaugcaaaacug............	91	1	seq
....ugugcaaaucUaugcaaaacuga...........	94	1	seq
....ugugcaaauccaugcaaaacuga...........	480	0	seq
....ugugcaaauccaugcaaaacugac..........	2	0	seq
....ugugcaaauccaugcaaaacugaU..........	11	1	seq
....ugugcaaauccaugcaaaacugaUu.........	1	1	seq
....gugcaaauccaugcaaaacug............	4	0	seq
